# Is Rising Obesity Causing a Secular (Age-Independent) Decline in Testosterone among American Men?

**DOI:** 10.1371/journal.pone.0076178

**Published:** 2013-10-16

**Authors:** Allan Mazur, Ronny Westerman, Ulrich Mueller

**Affiliations:** 1 Maxwell School, Syracuse University, Syracuse, New York, United States of America; 2 Institute for Medical Sociology and Social Medicine, Phillips University Marburg, Marburg, Germany; Oregon Health & Science University, United States of America

## Abstract

The testosterone of men in industrial societies peaks in their twenties and tends to decline with increasing age. Apart from this individual-level decline, there have been reports of a secular (age-independent population-level) decline in testosterone among American and Scandinavian men during the past few decades, possibly an indication of declining male reproductive health. It has been suggested that both declines in testosterone (individual-level and population-level) are due to increasing male obesity because men in industrial society tend to add body fat as they age, and overall rates of obesity are increasing. Using an unusually large and lengthy longitudinal dataset (991 US Air Force veterans examined in six cycles over 20 years), we investigate the relationship of obesity to individual and population-level declines in testosterone. Over twenty years of study, longitudinal decline in mean testosterone was at least twice what would be expected from cross-sectional estimates of the aging decline. Men who put on weight intensified their testosterone decline, some greatly so, but even among those who held their weight constant or lost weight during the study, mean testosterone declined 117 ng/dl (19%) over 20 years. We have not identified the reason for secular decline in testosterone, but we exclude increasing obesity as a sufficient or primary explanation, and we deny the supposition that men who avoid excessive weight will maintain their youthful levels of testosterone.

## Introduction

There have been scattered reports that mean testosterone (T) for men of a given age is decreasing among Americans [1] and Scandinavians [2]–[4]. The reliability and extent of, and reasons for, this “secular” decline are unknown. If valid, a secular decline in T is disquieting because it is congruent with other indications of degradation in male reproductive health such as falling sperm counts [5].

Male T is inversely correlated to body fat whether measured by body mass index (BMI) or by weight [6], [7]. There appears to be a causal connection behind this correlation, though whether it is one-way or bidirectional is uncertain. Obese men after gastric bypass surgery show higher T levels as their weight drops [8]–[10]. T gain (or loss) is also associated with weight loss (or gain) via nonsurgical means [11]. It is plausible that the secular (population level) decline in T, as well as declining T during an individual’s maturity, is accounted for by increasing fatness.

In industrial societies, T peaks when men are in their twenties and declines with continued aging [12], but see [13] for a possible exception in South Korea. Anthropologists report that this age-related decline in T is less or nonexistent among nonindustrial communities [14]–[16]. These nonindustrial results, based on small samples, must be regarded cautiously, but they do implicate industrial lifestyle in declining T. It is pertinent to ask, (1) How much of a man’s age-related decline in T may be due to his putting on weight?, and (2) Is there a secular (i.e., age-independent population-level) decline in T in industrial societies, and if so, is it attributable to rising rates of obesity?

Several cross-sectional and longitudinal studies find BMI and age to be independently related to T (e.g., [17], [18]), but there are still suggestions that controlling lifestyle, especially body weight, might greatly lessen if not eliminate the age-related decline in T (e.g., [11], [19], [20]). Unresolved is the degree to which declining T, whether at the population or individual level, is attributable to an increased tendency toward obesity. These matters are addressed here with an exceptionally long and large longitudinal dataset of 991 male U.S. Air Force veterans examined in six cycles over 20 years.

## Methods

### Ethics Statement

The US Institute of Medicine, which hold and controls access to the data used here, accepted the judgment of the Syracuse University IRB that access to de-identified existing data does not meet the definition of human subjects research as defined by the regulations and therefore does not require IRB approval and oversight.

### Data

The Air Force Health Study (AFHS) was intended to evaluate health effects of exposure to the dioxin-contaminated herbicide Agent Orange during the Vietnam War. It compared air and ground crewmen involved in wartime herbicide spraying (in Operation Ranch Hand) with matched Air Force veterans involved in other transport aircraft missions in Southeast Asia. “Ranch Hands” and designated comparison subjects were invited in 1982 for a baseline personal interview, physical examination, and psychological testing. At that time they ranged in age from 31 to 68 years. They were invited again in 1985, 1987, 1992, 1997, and 2002. There was little difference between men who participated in the physical examination and those who refused in terms of reported health status, medication use, and days lost from work. There were few discernible differences in the health of Ranch Hands and comparison subjects. Possibly type 2 diabetes is the most important dioxin-related health problem seen in the AFHS, but this is controversial [21]–[23].

Comparison subjects lost from the panel were replaced, and additional Ranch Hands were located and added, so that over 4,000 men participated in at least one cycle, with about 2,000 men in any single cycle. This study is based on 991 men who completed all six cycles and gave permission for their data to be used or who since died, in which case permission was not required for inclusion.

Compared to 1,881 men who completed all of the first four cycles [24], the 991 veterans analyzed here are about one year younger, slightly more educated (45% vs. 42% college graduates), slightly more likely to be married at each of the first four cycles (ca. 87% vs. ca. 84%), and less likely to be black (4% vs. 6%). Mean T levels (in ng/dl) for the samples of 991 and 1,881 differ by ≤10 ng/dl in each of the first four cycles. By these measures, the present sample of 991 men is not much different from the 1,881 who completed the first four cycles. The sample of 991 is biased at least in its subjects being sufficiently healthy to still be alive for the sixth cycle of the study. By 1992 27% of the 991 men in the sample were obese by the criterion BMI ≥ 30 kg/m^2^, and by 2002 35% were obese. These are similar to percentages of obesity reported by the US Centers for Disease Control [25] for the age-adjusted adult population (23% for 1988–94 and 35% for 2005–2008).

T was assayed in duplicate from morning blood samples taken before breakfast after an overnight fast. Quality control procedures required that when the coefficient of variation (CV) between duplicates was greater than 8%, assays were retaken. Mean CV between duplicates is about 5% [20]. About twenty of nearly 6,000 T values are either missing or are extraordinarily high (≥ 1400 ng/dl) or low (<100 ng/dl) for the sample as a whole and compared to the other T values recorded for the subject. Since these are most likely errors of measurement or recording, they are replaced by the mean of the subject’s two T values in adjacent cycles; for cycles 1 and 6, values ≥1400 are replaced by 1399. Means and standard deviations for T levels, none of them unusual, are shown by year of cycle in Table 1 with other descriptive statistics. T distribution in each cycle is positively skewed, as is typical. Skew  =  0.6 for the aggregate of T values for all men over all cycles. We opt to present results of statistical models in terms of raw T values for ease of interpretation. All results were confirmed by rerunning models with T transformed to remove skew.

**Table 1:** Mean (and Standard Deviation) or Percentage of Relevant Variables by Year of Cycle (n = 991).

	1982	1985	1987	1992	1997	2002
Testosterone (ng/dl)	638 (176)	611 (203)	535 (160)	520 (189)	440 (179)	431 (200)
Age (years)	43 (6.9)	46 (6.9)	48 (6.9)	53 (6.9)	58 (6.9)	63 (6.9)
Age range	31–63 Yrs.	34–66 Yrs.	36–68 Yrs.	41–73 Yrs.	46–78 Yrs.	51–83 Yrs.
>High school	54%	Same	Same	Same	Same	Same
Black	4%	Same	Same	Same	Same	Same
Ranch Hands	46%	Same	Same	Same	Same	Same
Married	88%	88%	83%	87%	85%	86%
BMI (kg/m^2^)	26.6 (3.5)	27.3 (3.8)	27.6 (3.9)	28.2 (4.1)	28.5 (4.5)	28.9 (4.3)
Obese(BMI≥ 30)	13%	19%	22%	27%	30%	35%
Weight (Kg.)	84.4 (12.2)	85.5 (12.6)	86.7 (13.0)	88.8 (13.9)	90.0 (15.1)	91.0 (14.8)

For graphical displays, subjects were roughly trichotomized into birth cohorts: 357 men born between 1918 and 1935, 337 men born between 1936 and 1944, and 297 men born from 1945 to 1950. Subjects were also trichotomized into percentage weight change over the 20 years of the study: 324 men had a loss or virtually no change from their cycle 1 weight, ranging from –30% to +2%; 336 men had medium gain, from +3% to 11%; and 331 men had high gain, from +12% to 54%. In order to graph mean T in a narrow age range across the twenty years of study, we also looked specifically at men of ages 51 to 60 at each cycle. This age range was selected because there was an adequate number of subjects in their fifties at every cycle. Error bars in the graphs are standard errors of the mean.

Data obtained from six examinations of the same man are not independent observations, so simple regression, which assumes independent observations, is not appropriate for analysis of data aggregated across all cycles. The preferred method is mixed-effects regression modeling for repeated measures of T with subject-level random effects. This was used to compare the effect of aging per se with the (secular) effect of cycle year on T, while controlling time-dependent covariates. Mixed effects models were run on Stata 12 IC by using the xtmixed command.

## Results

**Figure 1:**
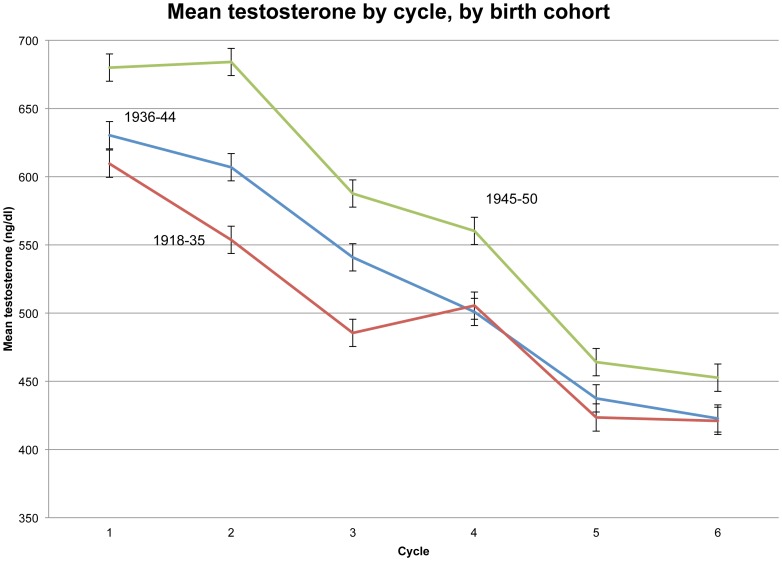
Mean testosterone by cycle, by birth cohort.

Figure 1 shows mean T’s across the six cycles for three birth cohorts, with error bars showing standard error of the mean. As expected, the youngest men, born 1945–50, had the highest mean T at each cycle. An unanticipated result is that the large birth cohort differences in T that are apparent in the first three cycles are minimal in the last three cycles, i.e., T varies little by age after cycle 3. Indeed, in cycles 4–6 there is barely any difference in mean T between the two oldest birth cohorts. Perhaps the oldest men with lowest T did not live to participate in later cycles, biasing the mean T of survivors upward, a possibility that cannot be tested with the data in hand.

### Secular Decline

Looking at any one cycle in Figure 1, we see a difference in mean T between the oldest and youngest birth cohorts (which differ in average age by roughly 20 years) of 100 ng/dl (in cycles 1–3) or less (in cycles 4–6). In the absence of any secular decline in T, we would expect from such cross-sectional estimates that simple aging would lower T about 100 ng/dl or less over the 20 years of the study. In fact, mean T for the 991 men fell from 638 ng/dl in 1982 to 431 ng/dl in 2002, a drop of 207 ng/dl. That is, longitudinally, T fell over twice as much as expected from cross-sectional estimates of the aging effect. Some factor other than simple aging must have intensified T decline over the 20-year course of the study. This replicates the age-independent secular decline previously reported for American and Scandinavian men.

To further explore the secular decline, for each cycle, T was regressed on age at that cycle. Table 2 shows for year of cycle the regression coefficient and constant (with p<.0001 indicated in bold). All coefficients are negative, indicating declining T with age. Coefficients for the first three cycles are relatively strong and highly significant (p<.0001), while those for the last three cycles are weaker and minimally or not significant. Consistent with Figure 1, cross-sectional age differences in T are small in the last three cycles. The constants, all highly significant, show decline from 1985 to 2002.

**Table 2:** Coefficient and constant for regression of testosterone on age, by year of cycle (n = 991).

	1982	1985	1987	1992	1997	2002
Coefficient (ng/dl)	–**4.12**	–**7.56**	–**6.19**	–2.60 (p = .003)	–2.07 (p = .01)	–1.68(n.s.)
Constant (ng/dl)	**814**	**958**	**834**	**659**	**561**	**536**

**Figure 2:**
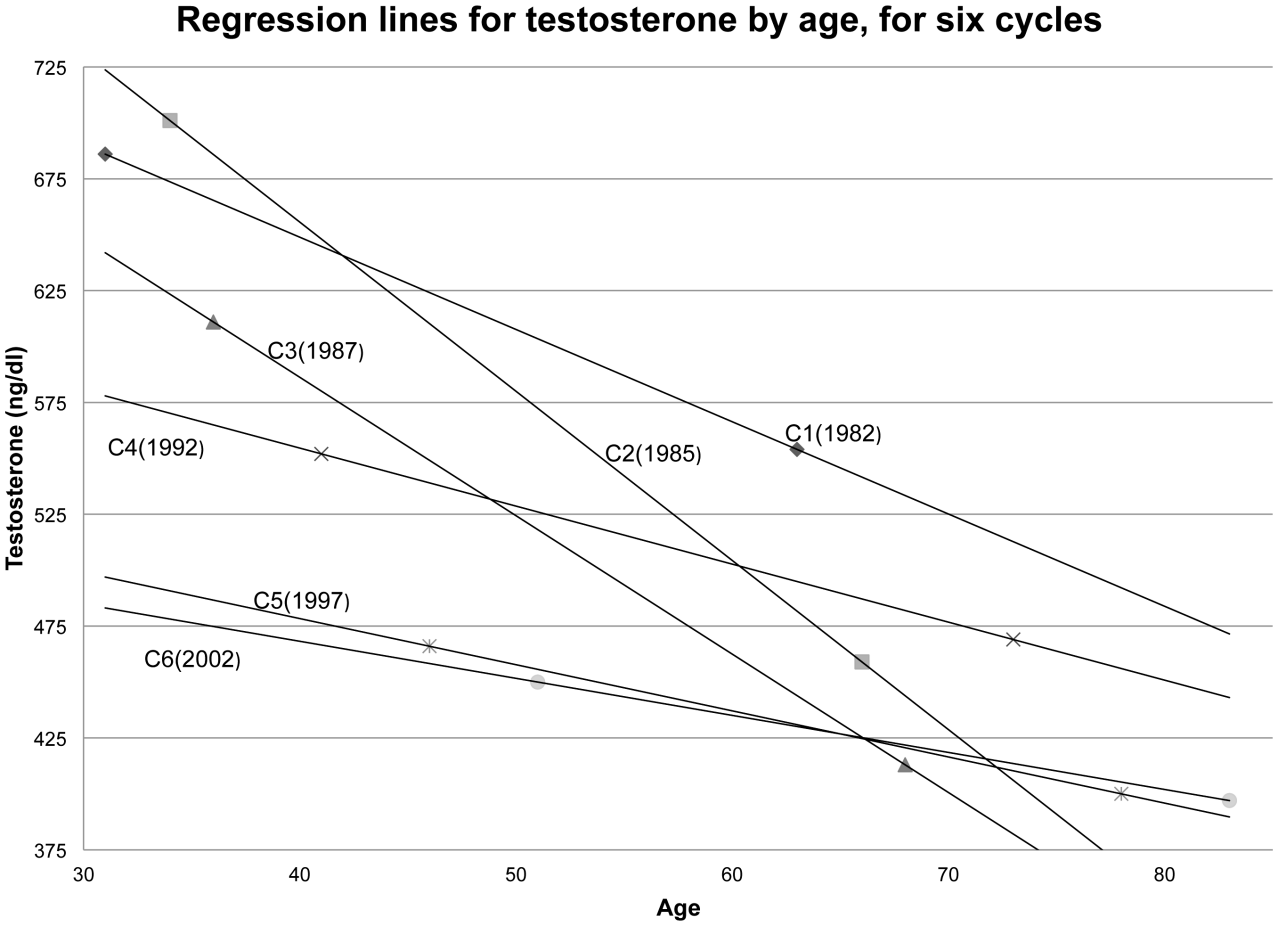
Regression lines for testosterone by age, for six cycles.

The regression lines for the six cycles are plotted in Figure 2. For unknown reasons (but consistent with Figure 1 and Table 2), regression lines for cycles 2 and 3 are notably steeper than for the other cycles, and the lines for cycles 4 through 6 are nearly flat. Nonetheless, the secular decline is manifest in a general pattern: At each age of measurement, T decreases from one cycle to the next (with some exceptions due to the steeper lines for cycles 2 and 3). The regression lines for cycles 5 and 6 (1997 and 2002) are nearly identical, possibly an indication that secular decline was no longer significant in these later years, though this might also be explained by older men with low T dying before cycle 6 and therefore being excluded from the sample.

### Putting on Weight

**Figure 3:**
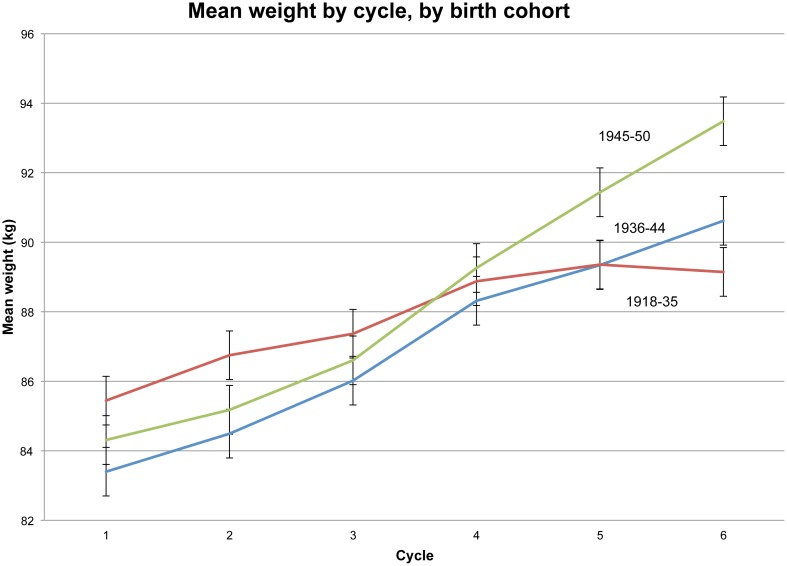
Mean weight by cycle, by birth cohort.

Mean weight rose for all age cohorts between 1982 and 2002 (Figure 3). The youngest men (born 1945–50), on average, continued to gain weight throughout the study, more so than men born a decade earlier (1936–44). The oldest cohort (born 1918–35) shows a strikingly different pattern, gaining weight from cycle 1 through cycle 5, and then losing weight in the last cycle. (This may explain why older men in the study had slightly higher T in cycle 6 than in cycle 5, as seen in Figure 2.) Men in the oldest cohort were mostly officers and college educated, likely making them more responsive to health counseling about losing excess weight. (The same patterns appear when BMI is substituted for weight.)

**Figure 4:**
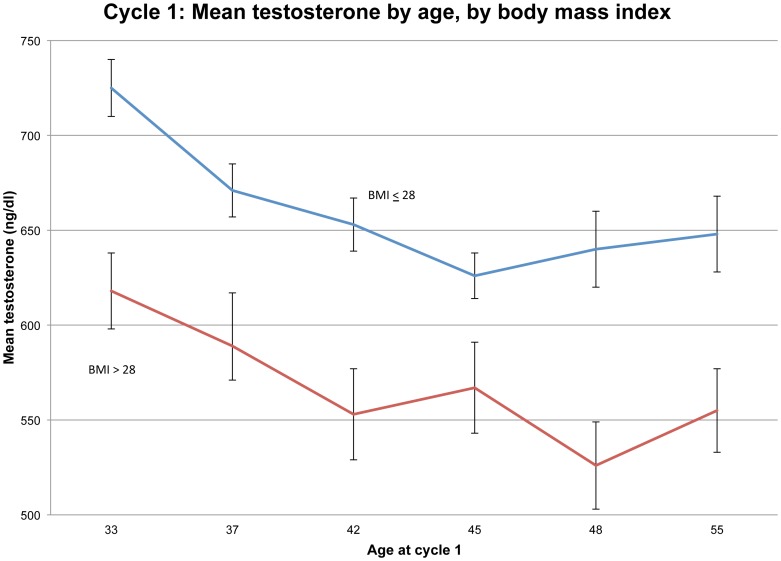
Cycle 1: Mean testosterone by age, by body mass index.

The well-known bearing of weight on T is shown for this dataset in Figure 4, where mean T in cycle 1 is plotted against age (in six categories) at cycle 1, while stratifying on BMI (≤28 vs. > 28). Other cycles produce similar patterns. These results lend credibility to the surmise that rising obesity is causing individual-level and population-level declines in T.

**Figure 5:**
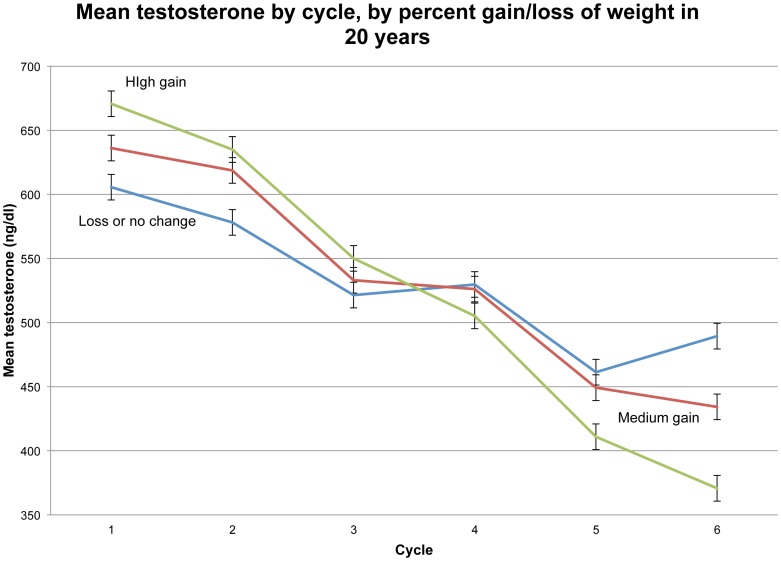
Mean testosterone by cycle, by percent gain/loss of weight in 20 years.

Mean T across cycles is again shown in Figure 5, but now the men are broken out according to the percentage of weight gain during the study. T differences among these weight-gain groups, about 100 ng/dl or less in any one cycle, are modest compared to the overall two-decade reduction in T of 207 ng/dl. Clearly weight gain cannot fully explain declining T. Indeed, the “loss or no change” group (i.e., the 324 men who lost as much as 30% of body weight and gained no more than 2% over the course of the study) still had a mean T reduction of 117 ng/dl (19%).

**Figure 6:**
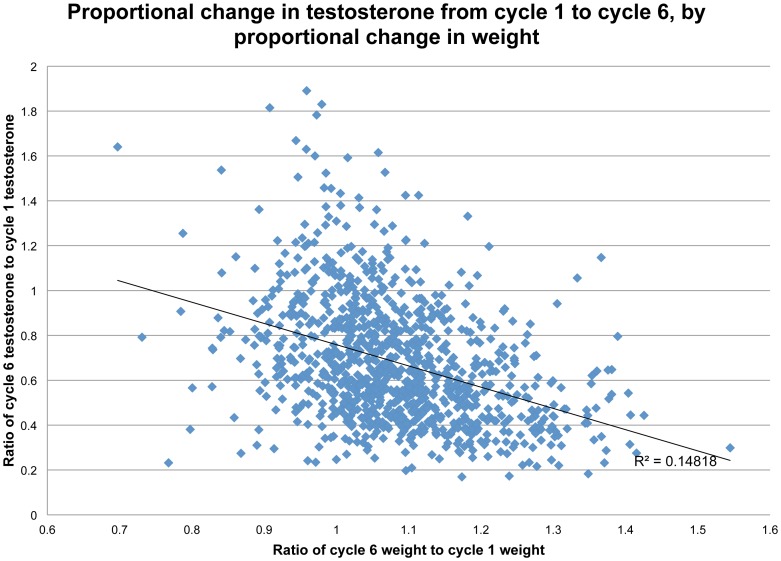
Proportional change in testosterone from cycle 1 to cycle 6, by proportional change in weight.

At the same time, it is clear that adding weight reduced T. The scattergram in Figure 6, showing percentage change in T (from cycle 1 to cycle 6) as a function of percentage gain in weight (across the same period), is fit with a linear regression line. Weight gain explains 16 percent of the variance in changing T (r = .41, p <.0001). The distribution of points shows that most men gained weight and lost T during the study, some considerably. However, among those who maintained their initial weight or lost weight, a fair portion had higher T at cycle 6 than at cycle 1.

Panel data permit a more detailed examination of this relationship, because we can compare each man’s cycle-to-cycle changes in weight (delta W) to his cycle-to-cycle changes in T (delta T). Table 3 is a Pearson correlation matrix of these changes between successive cycles. Correlations are generally small, never reaching 0.2 in absolute magnitude, however with so large a dataset, many are highly significant. Nonzero correlations are mostly negative, as expected, indicating that weight gain is associated with T loss. Correlations between delta W and deltaT during the same inter-cycle interval are highlighted in bold type. These bolded correlations have the highest absolute magnitudes in each row (or column), showing the simultaneity of changes in weight and T. DeltaT during one interval has virtually zero relationship to deltaW in a different interval. Unfortunately, the cycles are not sufficiently close in time for a profitable study of time lags between changes in T and weight.

**Table 3:** Pearson correlations between cycle-to-cycle changes in weight (delta W) and in testosterone (delta T).

	DeltaT12	DeltaT23	DeltaT34	DeltaT45	DeltaT56
DeltaW12	–0.16**	0.02	–0.04	–0.01	0.01
DeltaW23	0.05	–**0.09** *	–0.03	–0.02	–0.06
DeltaW34	0.03	–0.04	–0.17**	0.03	–0.04
DeltaW45	–0.04	0.01	0.01	–0.19**	0.01
DeltaW56	0.08*	–0.05	–0.06	0.06	–0.19**

* P <.01; **p<.0001.

### Estimating relative effects of age and cycle year

To take full advantage of the size and duration of this longitudinal study, while accommodating the nonindependence of data from each man’s six cycles, we used a mixed effects model to regress T on aging per se (measured by birth year) and cycle year. The model includes Ranch Hand (vs. control) as a constant covariate. Since T is known to drop in men after marriage [23], marital status (married or not) as well as body weight at each cycle are also controlled.

In total, the 991 men provide 5,946 data point over six cycles. Marital status was missing for 102 man-cycles, so the mixed effects model is based on 5,844 observations. The model is highly significant (p<.00001). Coefficients and their significance levels are shown in Table 4.

**Table 4:** Mixed-effects regression coefficients (ng/dl) and significance levels for T as dependent variable.

Independent variable	Coefficient	Significance level
Birth year	3.9	P <.00001
Cycle year	–8.9	P <.00001
Married (or not)	–41.9	P <.00001
Body weight (kg.)	–5.6	P <.00001
Ranch Hand (vs. Control)	4.2	P = 0.620

All coefficients except for Ranch Hand are highly significant. As expected, the coefficient for Birth year is positive, indicating that younger men have higher T. The coefficient for Cycle year is negative, showing the secular decline in T, independent of aging. Indeed, the Cycle year coefficient is more than twice the size of the Birth year coefficient, showing secular decline in T to be roughly twice as great as the decline in T due to aging per se. That is, for every year of aging (apart from secular decline), T drops 3.9 ng/dl, whereas for every passing calendar year (apart from aging), population-level T drops 8.9 ng/dl. Over the twenty years of study, secular decline accounts for a loss of 178 ng/dl of male T.

Married men (at each cycle) have nearly 42 ng/dl lower T than unwed men, a substantial and highly significant difference. However the percentage of married men at each cycle barely changes (Table 1), so changing marital status cannot explain secularly declining T. Apart from other factors, men lose nearly six ng/dl of T for every kilogram of body weight they put on. In other words, adding a kilogram accounts for more decrease in T than aging a year, but the secular decline in T during one year of calendar time is even greater than the effect of adding that kilogram.

**Figure 7:**
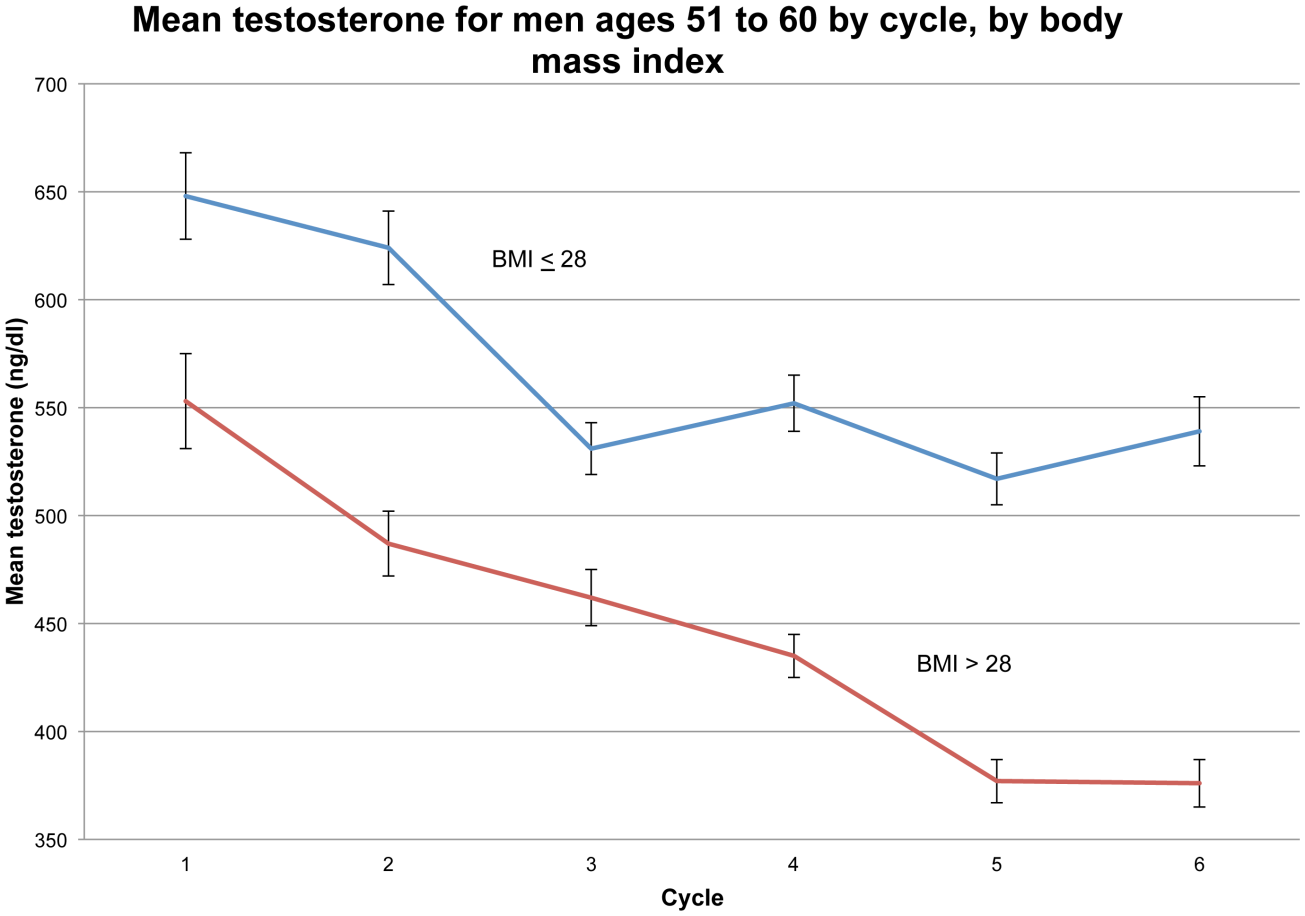
Mean testosterone of men ages 51 to 60 years by cycle, by body mass index.

To vividly illustrate the secular (age-independent) decline in T, we plot in Figure 7 the mean T of men in the age range 51–60 years at each cycle, also stratifying on BMI (≤28 vs. >28). From cycle to cycle, over the twenty years of study, we see mean T fall about 175 ng/dl for men in their fifties who are obese or nearly so (i.e., BMI > 28). For men in their fifties of moderate weight (BMI <28), mean T fell slightly over 100 ng/dl over the twenty years; most of this decline was during cycles 1–3.

## Discussion

We find a secular (population-level) decline in men’s T since the 1980s, as well as an individual-level decline in T with aging. Both occur independently of changing body weight and marital status. This corroborates the report by Travison et al. [18] of a population-level decline in T among American men and similar reports from Scandinavia. We have not identified a reason for the secular decline. In our sample, exposure to toxic Agent Orange during the Vietnam War was a potential explanation for diminished reproductive health, but we find no significant difference between the exposed Ranch Hands and control subjects. Possibly the magnitude of secular or individual decline in T would be moderated if we could control on cessation of smoking, increasing morbidity and use of medicines, but we lack this information. Prior reports of the magnitude of these effects suggest that they would not greatly alter our picture.

Becoming obese lowers T level, sometimes greatly so, but even among men who gained no weight or lost weight over the 20 years of study, mean T dropped 107 ng/dl (19%) by cycle 6 (Figure 5). We have seen from mixed effects modeling that secular decline alone accounts for a loss of 178 ng/dl of T over the 20 years of study, so it is plausible that men who maintained or lost body weight would have sustained or even raised their youthful levels of T if not for the population-level decline. We believe it is of highest importance to identify the cause of the secular decline in T.

We are puzzled by results in Figure 2, showing for each cycle the regression line for T on age. Our prior assumption was that these would be parallel (downward sloping) lines, lowering with each succeeding cycle. While this is true for cycles 1, 4, 5 and 6, the lines for cycle 2 (1985) and cycle 3 (1987) are anomalously steeper. Possibly some artifact in the data, perhaps due to unknown changes in assay procedures from one cycle to another, perturbed these two regression lines. On the other hand, it may be substantively true that in the cycle years 1985 and 1987, men in the AFHS tended to be in an age range when T declined particularly rapidly as a result of putting on weight, an effect that moderated, or nearly disappeared, as the men became elderly. We are unable to choose among these possibilities, which deserve further study.

Our sample is limited to men who lived long enough to complete all six cycles of the AFHS. At its conclusion, the oldest respondent was 83 years of age. Low T in men has been associated with shortened life span [26]. If so, our sample is biased in favor of men who had relatively high T in the later cycles. This could have produced an underestimate of the extent of secular decline. We think this possibility should be explored by researchers with full access to AFHS data.
